# Clinical efficacy of hyaluronate-containing embryo transfer medium in IVF/ICSI treatment cycles: a cohort study

**DOI:** 10.1093/hropen/hoab004

**Published:** 2021-03-03

**Authors:** Tope Adeniyi, Gregory Horne, Peter T Ruane, Daniel R Brison, Stephen A Roberts

**Affiliations:** 1 Department of Reproductive Medicine, Old Saint Mary’s Hospital, Manchester University NHS Foundation Trust, Manchester Academic Health Science Centre, Manchester, UK; 2 Division of Developmental Biology and Medicine, Maternal and Fetal Health Research Centre, School of Medical Sciences, Faculty of Biology Medicine and Health, University of Manchester, Manchester Academic Health Science Centre, Manchester, UK; 3 Maternal and Fetal Health Research Centre, Saint Mary’s Hospital, Manchester University NHS Foundation Trust, Manchester Academic Health Science Centre, Manchester, UK; 4 Division of Population Health, Health Services Research & Primary Care, School of Health Sciences, Faculty of Biology, Medicine and Health, Centre for Biostatistics, The University of Manchester, Manchester Academic Health Science Centre, Manchester, UK

**Keywords:** hyaluronate-containing medium, embryo transfer, clinical pregnancy, live birth event, embryo culture media, birthweight, safety

## Abstract

**STUDY QUESTION:**

Does the duration of embryo exposure to hyaluronic acid (HA) enriched medium improve the rate of live birth events (LBEs)?

**SUMMARY ANSWER:**

The use of embryo transfer (ET) medium rich in HA improves LBE (a singleton or twin live birth) regardless of the duration of exposure evaluated in this study, but does not alter gestation or birthweight (BW).

**WHAT IS KNOWN ALREADY:**

HA-enriched medium is routinely used for ET in ART to facilitate implantation, despite inconclusive evidence on safety and efficacy.

**STUDY DESIGN, SIZE, DURATION:**

A cohort study was performed evaluating clinical treatment outcomes before and after HA-enriched ET medium was introduced into routine clinical practice. In total, 3391 fresh ET procedures were performed using low HA and HA-rich medium in women undergoing publicly funded IVF/ICSI treatment cycles between May 2011 and April 2015 were included in this cohort study.

**PARTICIPANTS/MATERIALS, SETTING, METHODS:**

A total of 1018 ET performed using low HA medium were compared with 1198, and 1175 ET following exposure to HA-rich medium for 2–4 h (long HA exposure) or for 10–30 min (short HA exposure), respectively. A multiple logistic regression analysis was used to compare clinical outcomes including BW, gestational age and sex ratios between groups, whilst adjusting for patient age, previous attempt, incubator type and the number of embryos transferred.

**MAIN RESULTS AND THE ROLE OF CHANCE:**

The use of HA-rich medium for ET was positively and significantly associated with improved clinical pregnancy rate and LBE, for both exposure durations: long HA (odds ratio (OR) = 1.21, 95% CI: 0.99–1.48), short HA (OR = 1.32, 95% CI: 1.02–1.72) and pooled OR = 1.26, 95% CI: 1.03–1.54, relative to the use of low HA medium. A comparative analysis of the risks of early pregnancy loss following long HA exposure (OR = 0.76, 95% CI: 0.54–1.06), short HA exposure (OR = 0.84, 95% CI: 0.54–1.30) and late miscarriage (OR = 0.88, 95% CI: 0.51–1.53) (OR = 1.41, 95% CI 0.72–2.77), were lower and not statistically significant. Similarly, ordinary regression analysis of the differences in BW at both HA exposures; pooled OR = −0.9 (−117.1 to 115.3), and adjusted BW between both HA cohorts; pooled OR = −13.8 (−106.1 to 78.6) did not show any differences. However, a difference in gestational age (pooled OR −0.3 (−3.4 to 2.9)) and sex ratio (pooled OR 1.43 (0.95–2.15)) were observed but these were not statistically significant relative to low HA medium.

**LIMITATIONS, REASONS FOR CAUTION:**

The strength of a randomized treatment allocation was not available in this evaluation study, therefore effects of unmeasured or unknown confounding variables cannot be ruled out.

**WIDER IMPLICATIONS OF THE FINDINGS:**

The result of this large cohort study strengthens the case for using HA-rich medium routinely at transfer, while adding the important clinical information that duration of exposure may not be critical. The composition and effects of commercial IVF culture media on success rate and safety remains a major controversy despite increasing calls for transparency and evidence-based practice in ART. Nonetheless, the lack of differences in BW and gestational age observed in this study were reassuring. However, an appraisal of clinical outcomes and appropriate research investigations are required for the continuous evaluation of efficacy and safety of HA.

**STUDY FUNDING/COMPETING INTEREST(S):**

T.A. is funded by a Clinical Doctoral Research Fellowship (CDRF) grant (reference: ICA-CDRF-2015-01-068) from the National Institute for Health Research (NIHR). The views expressed in this publication are those of the authors and not necessarily those of the NHS, the National Institute for Health Research or the Department of Health. The authors declare no conflict of interest.

WHAT DOES THIS MEAN FOR PATIENTS?

Use of solutions containing hyaluronic acid (HA) during IVF treatment has been suggested to improve chances of a successful pregnancy and live birth rates following fertility treatment. Unfortunately, the way it improves treatment is unknown, and the evidence of its usefulness and safety is not yet conclusive. Despite this, it has continued to attract attention from patients, clinical practitioners and regulatory bodies and is routinely offered as an ‘add-on’ to fertility treatment. A commonly used medium of this type, with HA as the active ingredient, is called EmbryoGlue**^®^**, and this was introduced into routine use in our clinic with careful monitoring as part of an ongoing evidence-gathering project. Our first piece of research in the laboratory suggested that EmbryoGlue^®^ might not act by directly promoting embryo attachment to the womb, but by some other method.

In this clinical study, we introduced EmbryoGlue^®^ into our clinical practice for all couples and carefully evaluated the impact on pregnancy and live birth rates. Our findings are reassuring as they suggest that EmbryoGlue^®^ improves the chances of having a live birth, regardless of how long the embryo is exposed to it in the laboratory before transfer. Also, it may reduce the chances of patients experiencing early pregnancy loss or late miscarriage, and most importantly it does not affect the weight of the baby at birth. However, more research and continual evaluation are required to ensure that the use of HA transfer medium is safe and beneficial to infertile couples.

## Introduction

ART outcomes have improved since the birth of the world’s first IVF baby (Louise Brown) was reported in 1978 and an estimated 8 million babies have now been born from the application of various ART procedures (De Geyter *et al.*, 2018; Fauser, 2019; de Mouzon *et al.*, 2020). In the UK alone, more than 2% of all babies born are accounted for by ART with recent Human Fertilisation and Embryology Authority (HFEA) data showing a year on year increase in the number of treatment cycles (HFEA Figures and Trends: 2017 Report).

Modifications of ART culture techniques, *in vitro* embryo culture media compositions and the availability of a variety of commercially available single-step and sequential culture media have contributed to recent advances in ART (Hardarson *et al.*, 2015). The use of a dedicated embryo transfer (ET) medium with a known active ingredient hyaluronic acid (HA), a ubiquitous extracellular matrix molecule, has been evaluated in experimental (Gardner *et al.*, 1999; Berneau *et al.*, 2019) and to some extent in clinical settings (Simon *et al.*, 2003; Hazlett *et al.*, 2008) and randomized controlled trials (RCTs) (Valojerdi *et al.*, 2006; Urman *et al.*, 2008). The pioneering *in vitro* studies involving a mouse model suggested that HA promotes cell to cell and cell to matrix adhesions via its receptor CD44, which is expressed on the preimplantation embryo (Campbell *et al.*, 1995; Gardner *et al.*, 1999) and also on the endometrial stroma in mammals (Behzad *et al.*, 1994). The potentially beneficial effect of HA on embryo implantation was further evaluated in a Cochrane Review (Bontekoe *et al.*, 2010) which reported an increase of 8% in clinical pregnancy rate with adherence compounds used in ET media. A follow-up review (Bontekoe *et al.*, 2014) involving 3898 patients reported improvements in clinical pregnancy and live birth rate but with a higher risk of multiple pregnancies in HA-rich culture medium relative to media containing a lower, but functional concentration of HA. This evidence was graded as moderate, and the risk of adverse events was not significantly different between the different concentrations of HA. As a result of these clinical data, the use of HA-rich medium at the time of elective single embryo transfers to reduce the risks of multiple pregnancies has become widespread across assisted conception units (Harper *et al.*, 2017).

However, a recent RCT involving 581 cycles did not show any beneficial effect on implantation rate, but rather a higher birthweight (BW) was observed in the HA group, suggesting a reason for caution (Fancsovits *et al.*, 2015). With increasing calls for more evidence-based practice in modern reproductive medicine, the use of HA-rich culture medium and other ‘add-ons’ of IVF have come under increased scrutiny by several ART professional bodies; ESHRE, Association of Reproductive and Clinical Scientists, British Fertility Society, patient-led charities (Infertility Network UK) and governmental regulatory bodies. In particular HFEA, and the Medicines and Healthcare products Regulatory Agency now advocate greater transparency and safety amongst manufacturers and ART practitioners (Sunde *et al.*, 2016; Adamson *et al.*, 2018; Medical device alert 2018: MDA/2018/003; Armstrong *et al.*, 2019).

Therefore, in order to continually evaluate the use of EmbryoGlue^®^ in clinical practice, we carried out two parallel studies. The first, a pre-clinical study on human embryos donated to research, showed that EmbryoGlue^®^ did not increase the attachment of embryos to endometrial epithelium *in vitro* (Ruane *et al.*, 2020). In the second study, presented here, we evaluated clinical treatment outcomes in a cohort study before and after EmbryoGlue^®^ was introduced into routine clinical practice. Furthermore, we compared clinical treatment outcomes relative to the duration of exposure to EmbryoGlue^®^ and the risks of undesirable clinical outcomes, including altered gestation and BW, while controlling for potential confounding variables.

## Materials and methods

### Patient inclusion and study design

**Figure 1.**
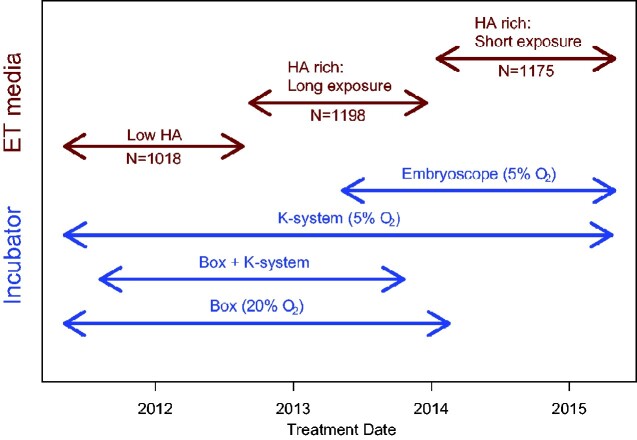
**Study design with changes in clinical practice.** The diagram shows patient allocation and corresponding changes in the type of incubator, O_2_ level and embryo transfer (ET) media. ‘Box + K-system’ refer to embryos that were initially cultured in the conventional style box incubator up to day 3 before transfer into K-systems incubator for blastocyst culture. HA, hyaluronic acid.

A total of 3391 consecutive patients undergoing publicly funded fresh IVF/ICSI treatment cycles at a UK National Health Service (NHS) assisted conception unit between May 2011 and April 2015 were evaluated in three different sequential cohorts, before and after the introduction of EmbryoGlue^®^ medium for ET. A total of 1018 ET procedures were conducted with the use of standard human embryo culture medium G^2+^ (G5-Series, Vitrolife, Gothenburg, Sweden), which contains a low level (0.125 mg/ml) of HA and recombinant human serum albumin (HSA) (10.0 mg/ml) between May 2011 and August 2012. The next 2373 ET procedures involved the use of HA-rich (0.5 mg/ml) and HSA (2.5 mg/ml) ET medium (EmbryoGlue^®^, Vitrolife, Gothenburg, Sweden). A total of 1198 ET procedures, conducted between September 2012 and December 2013, were with embryos pre-equilibrated in HA-rich medium for 2–4 h and 1175 ET procedures, conducted between January 2014 and April 2015, for 10–30 min, prior to the ET procedure (Fig. 1). These two different durations of pre-equilibration, 2–4 h and 10–30 min, are hereafter referred to as long HA exposure and short HA exposure durations. These are termed the ‘HA cohorts’.

All patients with fresh embryo(s) available for transfer on day 2, 3 or 5 were included in this study. Patients not progressing with a fresh ET owing to elective freeze-all or symptoms of ovarian hyperstimulation syndrome were therefore excluded. Treatment cycles associated with donor gametes or surgically retrieved sperm were excluded.

### Ethical approval

A service evaluation protocol was approved by the Research and Innovation team at the Manchester University NHS Foundation Trust (MFT). As this evaluation used only fully anonymized, routinely collected data, ethical approval was not required in accordance with the NHS Health Research Authority (HRA) guidelines (http://www.hra-decisiontools.org.uk/ethics/).

### Treatment

The ovarian stimulation, oocyte retrieval, embryo culture, grading and selection for cryopreservation, and transfer procedures have been previously described (Kalleas *et al.*, 2020; Castillo *et al.*, 2020a). Briefly, all patients were treated according to a standard long-down regulated or short ovarian stimulation protocol. When at least three follicles reached a mean diameter of 17 mm, hCG (Pregnyl 5000 IU, Organon Laboratories Ltd., Cambridge, UK) was administered. Luteal phase progesterone support was provided by administration of 400 mg of cyclogest pessaries (LD Collins Hampstead, UK) twice daily for 14 days.

### Fertilization check, embryo culture and transfer procedures

**Figure 2.**
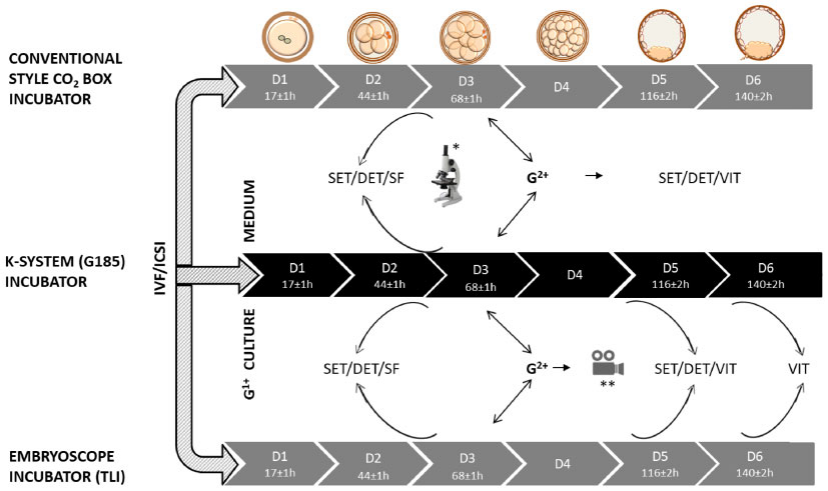
**A flow-chart illustrating the embryo culture system.** Fertilization checks were performed on day 1 (D1) between 16 and 18 h after insemination by conventional IVF or ICSI. Normal fertilized (2PN) oocytes were allocated to either a K-system (G185) or Embryoscope incubator based on space availability. Pronuclear stage embryos were cultured in G^1+^ medium until day 3 (D3). In cases where only one or two embryos are available, ET was performed on day 2. Embryos cultured in the conventional style box incubator and K-system were graded morphologically under a stereomicroscope at, approximately, post-insemination/injection on day 2 (44 ± 1 h), D3 (68 ± 1 h), D5 (116 ± 2 h) and D6 (140 ± 1 h). In the Embryoscope, morphological grading of embryos was performed at the same time-points by examining the time-lapse images. On D3, embryos were selected for replacement, for cryopreservation or for extended culture to the blastocyst stage: the latter were transferred to new culture dishes or Embryoscope slides containing G^2+^ medium for further culture to day 6. On day 5, the highest quality blastocysts were selected for either single embryo transfer (SET) or double embryo transfer (DET). Cryopreservation was by slow freezing (SF) for cleavage on D3 and vitrification (VIT) for blastocyst stage embryos. TLI, time lapse incubator **Embryoscope incubator timelapse assessment, *stereomicroscope assessment.

The fertilization check was performed on day 1, 16–18 h after insemination by ICSI or conventional IVF. All 2PN zygotes were cultured in groups in G^1+^ culture medium in a 60 mm Corning dish (Corning, NY, USA) and overlaid with 12 ml of Ovoil (Vitrolife, Gothenburg, Sweden) in a box incubator (HERAcell 240i, Thermo Scientific, Waltham, MA, USA) at 37°C and 5% CO_2_ until ET on day 2 or 3. The box incubator is similar to the conventional style CO_2_ incubator. All oocytes and embryos were assessed in accordance with the Istanbul Consensus (Balaban *et al.*, 2011). The ET procedure was performed using G^2+^ culture medium (G5-Series). Following the progressive introduction of the K-systems (BenchTop) and Embryoscope incubators, 2PN zygotes were non-selectively cultured individually in either a box or K-system incubator to day 3. Cases with at least three or more top quality embryos at the 8-cell stage were moved from G^1+^ culture medium into pre-equilibrated G^2+^ in the K-systems or Embryoscope incubators (embryoscope slide with 12 × 25 µl drops of G^2+^ overlaid with 1.2 ml of Ovoil) at 37°C, 5% O_2_ and 6% CO_2_ for blastocyst embryo culture (Fig. 2). Cycles with less than three top quality embryos at the 8-cell stage had ET on day 3, while day 2 ET was performed in all cases with only one or two embryos in culture following the day 2 embryo assessment.

Embryos that were unsuitable for cryopreservation or transfer remained in the embryo culture dish or Embryoscope slide, while embryos designated for ET were moved from G^1+^ or G^2+^ culture media into a dedicated ET dish. At ET, embryo(s) were moved from the 50 µl drop (G^2+^) into the 90 µl drop of EmbryoGlue^®^ for either a long or short duration and subsequently loaded into a Wallace Catheter (Smiths Medical Int., Hythe, UK) flushed twice with the aid of a 1 ml syringe (Becton Dickson, NJ, USA) as described by Critchlow *et al.* (1989) to ensure absence of toxicity using 3 ml of pre-equilibrated G^2+^ medium in a 5 ml falcon tube (Fisherscientific, UK). The catheter was placed into the uterine cavity under ultrasound guidance and, where necessary, an obturator was also used to direct the catheter.

### Outcome measures and statistical analysis

The primary outcome in this study was live birth event (LBE) defined as either a singleton or twin live birth. Secondary treatment outcomes were: biochemical pregnancy, clinical pregnancy (confirmed by ultrasound scan with the presence of a gestational sac and a foetal heartbeat at 6 weeks), multiple live births (the delivery of more than one baby), early pregnancy loss (a non-viable intra-uterine pregnancy with a gestational sac with or without a foetus or a heart-beat within 13 weeks of gestation) and late miscarriage (a spontaneous loss of a pregnancy between 14 and 24 weeks of gestation). Secondary infant outcomes were baby sex, gestational age at birth (GA), BW, gestation and sex-adjusted BW (BWadj) and BW centile. All BW data and gender information were patient reported. BWadj and BW centiles were calculated using the Gestation-Related Optimal Weight (GROW) formula based on standardized foetal growth data (http://www.gestation.net/); GA was computed as the number of days between ET and birth plus days in culture plus 14.

A pre-specified multiple logistic regression analysis was used to compare categorical outcomes between treatment groups. Analogous ordinary regression models were used for the continuous outcomes. These models adjusted for incubator type and O_2_ tension, maternal age as a four-category variable, number of embryos transferred (single embryo transfer or double embryo transfer), IVF or ICSI, number of IVF treatments (1st, 2nd or ≥3rd) and embryo culture duration (2, 3 or 5 days). HA treatment was included in the model as a three-category variable. Adjusted odds ratios (ORs) with associated 95% CI were calculated for each of the HA cohorts versus the low HA cohort. Further analogous models were fitted to: estimate the difference between the two HA treatments; and to estimate a pooled HA effect to simplify presentation and discussion. Additional exploratory analyses, including interactions between HA and embryo culture duration, were performed but these were not informative so are not presented here.

Analyses were conducted in the R statistical environment (v 3.6) as described by R Core Team (2019).

## Results

A total of 3391 fresh ET procedures were performed in women undergoing NHS-funded IVF/ICSI treatment cycles between May 2011 and April 2015. Patient and clinical characteristics were similar across the different study groups (Table I). However, the period of the study coincided with the introduction of bench-top (K-systems, G185) and timelapse (Embryoscope) incubators, and the phasing out of the conventional style box incubators (Fig. 1). The number of oocytes collected, fertilization and cleavage rate and surplus top-quality embryos were similar between the study cohorts. However, there was a modest increase in the proportion of blastocyst transfers in the later cohorts.

**Table I:** Patient and treatment characteristics.

Characteristics	Variables	G^2+^: Low HA	EmbryoGlue^®^: HA-rich medium	
Treatment and HA exposure time		*In situ* (untreated)	Long HA exposure (2–4 h)	Short HA exposure (10–30 min)
No. ET procedures		1018	1198	1175
Female age (years)	<35	631 (62.0%)	789 (65.9%)	754 (64.2%)
	35–37	212 (20.8%)	228 (19.0%)	233 (19.8%)
	38–39	144 (14.1%)	152 (12.7%)	154 (13.1%)
	40–42	31 (3.0%)	29 (2.4%)	34 (2.9%)
No. prior IVF/ICSI cycles	1st	663 (65.1%)	851 (71.0%)	878 (74.7%)
	2nd	296 (29.1%)	270 (22.5%)	251 (21.4%)
	3rd +	59 (5.8%)	77 (6.4%)	46 (3.9%)
Type of treatment	ICSI	625 (61.4%)	708 (59.1%)	684 (58.2%)
	IVF	393 (38.6%)	490 (40.9%)	491 (41.8%)
Type of incubator	Box	336 (33.0%)	340 (28.4%)	3 (0.3%)
	Box + K-system	256 (25.1%)	156 (13.0%)	0 (0.0%)
	K-system	426 (41.8%)	613 (51.2%)	449 (38.2%)
	Embryoscope	0 (0.0%)	89 (7.4%)	723 (61.5%)
Embryo stage at ET	Day 2	241 (23.7%)	264 (22.0%)	287 (24.4%)
	Day 3	571 (56.1%)	545 (45.5%)	496 (42.2%)
	Day 5	206 (20.2%)	389 (32.5%)	392 (33.4%)
No. embryos transferred	SET	388 (38.1%)	450 (37.6%)	457 (38.9%)
	DET	630 (61.9%)	748 (62.4%)	718 (61.1%)

DET, double embryo transfer; ET, embryo transfer; HA, hyaluronic acid; SET, single embryo transfer.

The use of HA-rich medium for ET was positively associated with improved LBE, at both exposure durations (long and short HA exposure; pooled OR = 1.26, 95% CI: 1.03–1.54), relative to the use of G^2+^ culture medium containing a low concentration of HA (low HA; Table II). Similarly, a positive association of HA-rich medium was observed with clinical pregnancy rate regardless of the duration of exposure (pooled OR = 1.24; 1.02:1.50).

**Table II:** Effect of HA on pregnancy outcomes.^**a**^

Outcome	Cohort	Number (%)	OR^a^ (95% CI)	*P* ^a^
LBE^b^	Low HA	224 (22.0%)	**Reference**	
	Long HA exposure	341 (28.5%)	1.26 (1.02:1.55)	0.029
	Short HA exposure	391 (33.3%)	1.28 (0.97:1.67)	0.078
	HA pooled effect	732 (30.8%)	1.26 (1.03:1.54)	0.023
Multiple birth^c^	Low HA	43 (4.2%)	**Reference**	
	Long HA exposure	66 (5.5%)	1.08 (0.71:1.64)	0.73
	Short HA exposure	80 (6.8%)	0.95 (0.56:1.60)	0.84
	HA pooled effect	146 (6.2%)	1.04 (0.69:1.57)	0.84
Biochemical pregnancy^d^	Low HA	337 (33.1%)	**Reference**	
	Long HA exposure	447 (37.3%)	1.08 (0.90:1.31)	0.39
	Short HA exposure	519 (44.2%)	1.20 (0.94:1.54)	0.15
	HA pooled effect	966 (40.7%)	1.11 (0.93:1.33)	0.26
Clinical pregnancy^e^	Low HA	252 (24.8%)	**Reference**	
	Long HA exposure	368 (30.7%)	1.21 (0.99:1.48)	0.059
	Short HA exposure	434 (36.9%)	1.32 (1.02:1.72)	0.036
	HA pooled effect	802 (33.8%)	1.24 (1.02:1.50)	0.031
Early pregnancy loss^f^	Low HA	85 (8.3%)	**Reference**	
	Long HA exposure	78 (6.5%)	0.76 (0.54:1.06)	0.11
	Short HA exposure	85 (7.2%)	0.84 (0.54:1.30)	0.42
	HA pooled effect	163 (6.9%)	0.78 (0.57:1.07)	0.12
Late miscarriage^g^	Low HA	28 (2.8%)	**Reference**	
	Long HA exposure	28 (2.3%)	0.88 (0.51:1.53)	0.65
	Short HA exposure	40 (3.4%)	1.41 (0.72:2.77)	0.31
	HA pooled effect	68 (2.9%)	1.00 (0.60:1.67)	0.99

The data show clinical outcomes following ET procedures and the effects of long and short exposures to HA-rich culture medium (EmbryoGlue^®^).

^a^ Adjusted LBE, multiple births, biochemical pregnancy, clinical pregnancy, early pregnancy loss and late miscarriage.

^b^ Deliveries that resulted in at least one baby.

^c^ Deliveries with more than one baby.

^d^ Positive βhCG test without a pregnancy on ultrasound scan, 3 weeks post-ET.

^e^ Pregnancies confirmed by ultrasound scan with the presence of a gestational sac and a foetal heartbeat at 12 6/7 weeks.

^f^ Losses occurring up to 13 weeks of gestation.

^g^ Losses between 14 and 24 weeks of gestation.

HA, hyaluronic acid; LBE, live birth event; OR, odds ratio.

The risk of early pregnancy loss tended to be reduced with the use of HA-rich culture medium relative to the use of low HA, independent of the duration of exposure: long HA exposure (OR = 0.76, 95% CI: 0.54–1.06), short HA exposure (OR = 0.84, 95% CI: 0.54–1.30), however, this did not reach statistical significance. Similarly, the risk of late miscarriage tended to be different in long HA exposure (OR = 0.88, 95% CI: 0.51–1.53) compared to short HA exposure (OR = 1.41, 95% CI 0.72–2.77); however, these were not statistically significant relative to low HA after allowing for multiple testing (Table II). The risks of patients experiencing adverse clinical outcomes (multiple-births and biochemical pregnancies) were marginally and not significantly associated with the use of HA-rich medium and this was independent of the duration of exposure prior to ET.

In the regression models (Supplementary Table SI), all the included factors (age, number of previous cycles and number and stage at which embryos were transferred) showed the expected associations with LBE (Valojerdi *et al.*, 2006; Urman *et al.*, 2008; Bontekoe *et al.*, 2014). Of note, we also included incubator type, based on our previous finding of an association between Embryoscope and LBE (Kalleas *et al.*, 2020), and we are able to confirm that positive association here.

Ordinary regression analysis of offspring BW, GA, BWadj and BW centiles did not show any differences between the use of low HA and HA-rich media at either time exposure (Table III). A difference was observed in gender distribution between the groups, but this did not reach statistical significance in this analysis.

**Table III:** Singleton offspring sex, birthweight and gestational age.

Outcome	Cohort	N (%)		OR^a^	*P* ^a^
^*^Sex ratio (male %)	Low HA	86 (51.2%)		**Reference**	
	Long HA exposure	150 (57.5%)		1.39 (0.92:2.12)	0.12
	Short HA exposure	163 (53.4%)		1.55 (0.91:2.64)	0.11
	HA pooled effect	313 (55.3%)		1.43 (0.95:2.15)	0.09

		**N**	**Mean (SD)**	**Difference^a^**	***P* ^a^**

Birthweight (g)	Low HA	168	3265 (607)	**Reference**	
	Long HA exposure	261	3241 (586)	−8.2 (−127.1:110.7)	0.89
	Short HA exposure	305	3280 (552)	27.3 (−123.8:178.4)	0.72
	HA pooled effect	566	3262 (568)	−0.9 (−117.1:115.3)	0.99
Gestational age (weeks)	Low HA	168	275 (16)	**Reference**	
	Long HA exposure	261	273 (18)	−0.9 (−4.1:2.4)	0.60
	Short HA exposure	305	275 (13)	2.1 (−2.0:6.3)	0.31
	HA pooled effect	566	274 (16)	−0.3 (−3.4:2.9)	0.88
GROW-adjusted birthweight (g)	Low HA	168	3412 (520)	**Reference**	
	Long HA exposure	261	3406 (420)	−4.0 (−98.5:90.6)	0.93
	Short HA exposure	305	3397 (454)	−51.5 (−171.6:68.6)	0.40
	HA pooled effect	566	3401 (438)	−13.8 (−106.1:78.6)	0.77
GROW birthweight centile	Low HA	165	40 (30)	**Reference**	
	Long HA exposure	256	39 (28)	−1.1 (−7.1:4.9)	0.71
	Short HA exposure	302	39 (28)	−4.6 (−12.1:3.0)	0.24
	HA pooled effect	558	39 (28)	−1.8 (−7.7:4.0)	0.54

^a^ OR adjusted simultaneously for all the known potential confounders (patient age, previous attempts, ICSI, SET/DET, blastocyst transfer and crucially incubation system).

* Reported for singleton births.

DET, double embryo transfer; HA, hyaluronic acid; OR, odds ratio; SET, single embryo transfer.

## Discussion

This study was undertaken as the second of a two-part clinical service evaluation of the use of HA-rich medium (EmbryoGlue^®^) for ET (Ruane *et al.*, 2020).

HA-rich medium showed a significant increase in the odds of achieving a clinical pregnancy and live birth relative to using low HA medium (G^2+^) for ET, with similar effects seen for both durations (long and short HA exposures). This observation is consistent with most previous studies evaluating the efficacies of so-called ‘adherence compounds’ in ART (Valojerdi *et al.*, 2006; Friedleri *et al.*, 2007; Urman *et al.*, 2008; Nakagawa *et al.*, 2012; Bontekoe *et al.*, 2014). Conversely, other studies (Simon *et al.*, 2003; Hambiliki *et al.*, 2010; Fancsovits *et al.*, 2015) failed to identify any statistically significant improvement in clinical pregnancy, implantation and LBE rates following the use of HA-rich medium. The reasons for this discrepancy are unclear, and demonstrate the need for ongoing service evaluation of the use of such compounds for ET.

Unlike most previous studies, our cohort study design provided additional insights into the efficacy of HA-rich medium in two different durations of exposure. We also evaluated the risks of early pregnancy loss and adverse treatment outcomes, including multiple births, based on the duration of exposure to HA-rich medium. We were unable to show an impact of HA-rich medium, for either duration of exposure, on any of these outcome measures. The small trends, towards increased risk of multiple births and biochemical pregnancies with the use of HA-rich medium, although not reaching statistical significance in the present study, are consistent with other studies (Chen *et al.*, 2001; Friedleri *et al.*, 2007; Bontekoe *et al.*, 2014; Nishihara and Morimoto, 2017). Finally, we also analysed BW and GA as part of our safety evaluation of the impact of ART and, reassuringly, saw no differences in any measure with HA-rich medium for either exposure time.

Importantly, for a longitudinal cohort study such as this during which other aspects of ART changed, our analysis models also provided estimates of the effects of different types of incubators and O_2_ concentrations (conventional style box incubator at 37°C and 6% CO_2_, atmospheric oxygen; K-system and Embryoscope (time lapse incubator, TLI) at 37°C, 6% CO_2_ and 5% Oxygen at) on LBE. In particular, the use of TLI and blastocyst stage ETs had a substantive effect, with TLI showing a statistically significant association with LBE relative to K-system, conventional style box incubator and a combination of conventional style box incubator and K-system. This observation was consistent with our previous clinical study (Kalleas *et al.*, 2020), which suggests that the undisturbed culture environment, use of a single-step embryo culture medium at low oxygen tension (5% O_2_,) and the individual culture well of the Embryoscope may be linked with improvements in LBE. These findings were consistent with other studies which also compared human embryo development in different culture environments, bench-top and Embryoscope incubators (Barrie *et al.*, 2017; Alhelou *et al.*, 2018; Sciorio *et al.*, 2018). The analysis carefully controlled for the known confounding due to all known changes and variation in clinical practice in incubation (incubator type and O_2_ levels), treatment type, stage of transfer and number of embryos transferred, and no other relevant changes in practice or patient population were identified over the study period. Nevertheless, our cohort study design did not have the benefit of random treatment allocation and therefore effects of residual or unmeasured confounding factors caused by drifts in practice or patient population over time (Castillo *et al.*, 2019b) cannot be ruled out.

The impact of commercially available human embryo culture media (Brison *et al.*, 2013) on the long-term health of ART offspring remains controversial (Dumoulin *et al.*, 2010; Chronopoulou and Harper, 2015; Kleijkers *et al.*, 2015; Zandstra *et al.*, 2015) and warrants continuous monitoring (Kleijkers *et al.*, 2014; Hann *et al.*, 2018; Castillo *et al.*, 2020a). In this cohort study, it was reassuring that we saw no difference in gestation and BW with HA exposure. However, Fancsovits *et al.* (2015) did observe a difference in BW (*P* = 0.001) with HA, and so this should remain an area for further investigation. Understanding the impact of culture media is complicated by the fact that we lack full disclosure of media composition, despite repeated calls for this (Biggers and Racowsky, 2002; Sunde *et al.*, 2016; Morbeck *et al.*, 2017). For EmbryoGlue^®^, the 4-fold increase in the concentration of HA relative to 0.125 mg/ml in G^2+^ appears to be the critical difference between the two culture media, although the concentration of recombinant HSA is 4-fold lower in EmbryoGlue^®^ medium compared with 10 mg/ml in G^2+^. Furthermore, we do not know the molecular weight profile of the HA in commercial EmbryoGlue^®^, and this in turn has significant implications for our understanding (Fouladi-Nashta *et al.*, 2017; Ruane *et al.*, 2020). The impact of other unknown components of these commercial embryo culture media may have significant influence on the mechanism of action of HA; while this remain unknown to us, our aim was to assess the efficacy of specific commercially available embryo culture media containing HA.

The mechanism of action of HA at implantation remains yet to be fully elucidated, despite investigations in both human and animal experimental models (Fouladi-Nashta *et al.*, 2017). HA affects cellular processes, including adhesion (Vigetti *et al.*, 2014), as well as promoting embryo development and endometrial decidualization (Urman *et al.*, 2008; Block *et al.*, 2009), and has been proposed to promote blastocyst attachment to the endometrial epithelium (Aplin and Kimber, 2004). Our recent experimental study described an *in vitro* model of embryo implantation and showed a trend towards protracted attachment kinetics of blastocysts exposed to HA-rich ET medium (EmbryoGlue^®^) (Ruane *et al.*, 2020). We further manipulated embryo-endometrial interactions *in vitro* using mouse blastocysts and found that degradation of HA at the embryo-endometrial interface significantly enhanced the kinetics of blastocyst attachment (Berneau *et al.*, 2019). Alongside data from sheep (Marei *et al.*, 2017), these mechanistic studies suggest that HA may actually attenuate blastocyst attachment to the endometrial epithelium at implantation. There is evidence that pre-attachment interactions with the endometrial epithelium stimulate subsequent embryo invasion (Ruane *et al.*, 2017) and so attenuated attachment may actually promote implantation success. Moreover, we and others speculate that HA may be acting as an embryonic growth factor, with at least the lower MW HA able to penetrate the zona pellucida (Campbell *et al.*, 1995; Legge, 1995; Fouladi-Nashta *et al.*, 2017). Such an embryo trophic mechanism could underlie the increased implantation seen when HA transfer medium is used in cleavage stage transfer. Importantly, human embryos express HA metabolism and receptor genes (HAS1-3, HYAL1-3, CD44 and HMMR) at all stages of preimplantation development, raising the prospect that HA may be active at different stages of embryonic development.

Clinical evidence suggesting the efficacy of HA-rich culture medium for ET has encouraged its routine use in ART (Bontekoe, *et al.*, 2010, 2014). Nonetheless, the standard of evidence reached has not been universally accepted: as an example, the UK HFEA traffic light system for add-ons gives HA-rich medium an amber rating, meaning that more research is required before routine use is recommended (https://www.hfea.gov.uk/treatments/treatment-add-ons/hyaluronate-enriched-medium/).

In this study, we report on a controlled introduction of a dedicated HA-rich medium (EmbryoGlue^®^) into routine clinical practice as an alternative to G^2+^, over an extended duration of study and exposures, as part of ongoing clinical service evaluation of safety and for continuous quality improvement purposes. Our data add to the clinical evidence that HA improves LBE. They further show that this effect is independent of exposure time, which is important evidence for informing clinical protocols. Finally, the study provides reassurance that HA-rich medium is not associated with adverse outcomes including pregnancy loss, multiple birth or altered gestation and BW. Additional investigations into the mechanisms of action of HA and its’ role in enhancing implantation rates continue to be warranted.

## Supplementary data

Supplementary data are available at *Human Reproduction Open* online.

## Supplementary Material

hoab004_Supplementary_Data
