# Supplementary material for: Clinical efficacy of hyaluronate-containing embryo transfer medium in IVF/ICSI treatment cycles: a cohort study
Source: Hum Reprod Open. 2021 Mar 3;2021(1):hoab004. doi: 10.1093/hropen/hoab004 (PMC7937422; doi:10.1093/hropen/hoab004)
Supplement: hoab004_Supplementary_Data [file hoab004_supplementary_data.docx]

**Supplementary Table SI** Full results of the multiple logistic regression analysis for live birth events.

| Treatment variables | OR (95% CI) | P |
| --- | --- | --- |
| Female age (yrs) | Reference: <35 years | |
| Category 1: 35-37 | 0.83 (0.68:1.02) | 0.071 |
| Category 2: 38-39 | 0.60 (0.46:0.77) | <0.001 |
| Category 3: 40-42 | 0.59 (0.34:1.01) | 0.054 |
|  | | |
| No. of attempts | Reference: 1^st^ attempt | |
| 2^nd^ attempt | 0.92 (0.76:1.10) | 0.35 |
| ≥ 3^rd^ attempts | 0.64 (0.43:0.94) | 0.024 |
|  |  |  |
| Treatment Type IVF versus ICSI | 0.99 (0.85:1.16) | 0.93 |
|  | | |
| Type of incubator | Reference: box incubator | |
| K-system (BenchTop) | 1.22 (0.96:1.55) | 0.11 |
| Box + K-system (BenchTop) | 1.22 (0.88:1.68) | 0.24 |
| Embryoscope (TLI) | 1.69 (1.23:2.33) | 0.001 |
|  | | |
| Embryo stage at transfer | Reference: Day 2 | |
| Day 3 ET | 1.81 (1.44:2.28) | <0.001 |
| Day 5 ET | 2.91 (2.26:3.74) | <0.001 |
|  |  |  |
| Total embryo transferred |  |  |
| DET versus SET | 1.53 (1.27:1.83) | <0.001 |
|  | | |
| Duration of HA exposure | Reference: G^2+^ | |
| Long exposure | 1.28 (1.04:1.57) | 0.019 |
| Short exposure | 1.25 (0.96:1.64) | 0.099 |

Odds ratios (OR) and significance levels adjusted for all the variables listed and are expressed relative to the corresponding reference.

HA: hyaluronic acid

TLI: timelapse incubator

ET: embryo transfer

SET: single embryo transfer

DET: double embryo transfer
